# Breastfeeding mothers’ experiences with community physicians in Israel: a qualitative study

**DOI:** 10.1186/s13006-022-00506-4

**Published:** 2022-08-30

**Authors:** Elia Blitman, Aya Biderman, Ilan Yehoshua, Limor Adler

**Affiliations:** 1 Department of Family Medicine, Maccabi Healthcare Services, Tel Aviv, Israel; 2 Department of Family Medicine and Siaal Research Center for Family Medicine and Primary Care, Faculty of Health Sciences, Ben-Gurion University of the Negev, Beer-Sheva, Israel; 3 Clalit Health Services, Southern District, Beer-Sheva, Israel; 4 Department of Family Medicine, Sackler Faculty of Medicine, Tel Aviv University, Tel Aviv, Israel

**Keywords:** Breastfeeding, Primary care, Qualitative research, Ante-natal care, Postpartum care

## Abstract

**Background:**

The guidelines of all leading professional organizations recommend providing adequate support and education regarding breastfeeding; yet many mothers feel that they receive inadequate information from their health care providers in the primary care setting. This is in line with studies that demonstrate that physicians’ knowledge about breastfeeding is lacking. The aim of this study was to expand our understanding of the breastfeeding-related experiences of mothers with primary care physicians (PCPs).

**Methods:**

In this qualitative study, we interviewed breastfeeding mothers in Israel in the first six months after delivery. The interviews were conducted between December 2020 and May 2021. We used thematic analysis to explore women’s attitudes and experiences with their PCPs regarding breastfeeding concerns. All authors read the transcribed interviews and independently marked statements regarding breastfeeding. Then, in a joint process, codes, subthemes and themes were defined. Each subtheme was backed up with a quote from the interviews.

**Results:**

We interviewed 13 women aged 24 to 37. We identified four main themes. The first of these was physicians’ inconsistent attitudes toward breastfeeding. Some were indifferent, while others related to breastfeeding solely in the context of infant development. Some were supportive, while others opposed breastfeeding. Several women revealed physicians’ inappropriate and disturbing attitudes to breastfeeding. The second theme was physicians’ lack of knowledge regarding medical treatment for breastfeeding issues. This theme included lack of knowledge, incorrect treatment of breastfeeding problems, and contradictions among HCPs. The third was mothers’ preference for alternative resources, including individualized breastfeeding counselling, maternity and childcare nurses, mothers’ groups (in person or online), and family and friends over medical treatment for breastfeeding problems. The fourth theme involved mothers’ suggestions for PCPs, which highlighted the importance of communication, prenatal physician-initiated dialogue on breastfeeding, expanding professional knowledge on breastfeeding, and increasing the availability of treatment for breastfeeding problems.

**Conclusion:**

The women in this study reported unsatisfactory breastfeeding support by PCPs and incorrect or inadequate treatment of medical problems related to breastfeeding. They also felt they had no medical experts to approach with breastfeeding-related problems. We believe that physicians should expand their knowledge on breastfeeding medicine so that they can provide comprehensive patient-centered treatment to both mothers and infants. Education programs for improving knowledge and skills in breastfeeding issues should be implemented throughout the medical training.

## Background

The guidelines of all leading professional organizations (of gynecologists, pediatricians, and family physicians) recommend providing adequate support for breastfeeding mothers and structured antenatal and postpartum breastfeeding education [1–3]. Nevertheless, many mothers feel that they receive inadequate information from their health care providers (HCPs) in the primary care setting [4].

In a systematic review of qualitative studies regarding infant feeding, one of the main points that emerged was that parents’ expectations regarding HCPs’ provision of individualized instructions regarding infant feeding were not always met. Many parents reported that the support and advice they had received was inadequate, nonexistent, inconsistent, or contradictory [4].

Despite the evidence-based medical benefits of breastfeeding, an Israeli study of 2,114 mothers showed that at age six months, only 22.5% of Jewish mothers and 12.3% of Arab mothers were exclusive breastfeeding their babies (true exclusive breastfeeding, since birth) [5]. In the same study, women revealed that among the factors that influenced their decision to breastfeed, their physician’s opinion was dominant [5]. And yet, two to six months after giving birth, 90% of the breastfeeding women stated that they had not received any breastfeeding support from their physicians and only a minority considered their physicians reliable sources of information in this realm [5].

### The Israeli healthcare system

The Israeli healthcare system is based on universal coverage, which is financed through taxes and provided by four healthcare maintenance organizations (HMOs) [6]. During pregnancy, most women are cared for by a gynecologist, a nurse, and a family physician. Women are advised to visit their gynecologists six weeks postpartum. Parents attend mother and child clinics for routine visits 10 days after delivery, and again at one, two, four, six, nine, and 12 months after delivery. They may also visit their family physician or their child’s pediatrician when they choose to. Breastfeeding consultations by qualified nurses (many of whom are also international Board-Certified Lactation Consultants) are offered through mother and child clinics or home visits by private nurses.

### Study aims

This qualitative study aimed to expand our understanding of the experiences of breastfeeding mothers with primary care physicians (PCPs) regarding breastfeeding. 

The results of this study can help PCPs to better recognize and understand breastfeeding women’s medical needs and difficulties and meet their demands and expectations.

## Methods

### Study design and setting

We conducted a qualitative study to shed light on the experiences of breastfeeding mothers with PCPs regarding breastfeeding in the ambulatory setting in the first six months after delivery. We interviewed women from Maccabi Healthcare Services (MHS), the second-largest HMO in Israel, which covers more than 2.6 million patients nationwide and approximately 40,000 live births each year. The local ethics committee (IRB) of MHS approved this study (ID 0130–19-MHS).

### Participants

The study participants were women with children aged 0 to 6 months at the time of the interview who had any experience with breastfeeding their current baby and who had had any contact with physicians in the ambulatory setting since delivery. All participants were Jewish and lived in the southern district of Israel. Their ages ranged from 24 to 37, with a median age of 33. Children’s age ranged from two to six months (Table 1).

**Table 1:** Participants’ characteristics

Study participant	Age (years)	Child’s age (months)	Number of children in the family
1	35	6	2
2	32	6	2
3	32	2	4
4	36	6	3
5	37	2	1
6	37	3	3
7	33	4	1
8	33	6	1
9	35	5	1
10	32	3	2
11	37	3	1
12	30	6	1
13	24	2	1

### Data collection

We recruited women through MHS’s mother and child nurses, breastfeeding counsellors, and social media. Nurses suggested to mothers that they could take part in this study. Women interested in participating in the study provided contact details for the lead author (EB). When women found out about the study from social media, they contacted EB and expressed their willingness to participate in it. Prior to the interviews, we sent each participant an informed consent form to sign. EB and LA interviewed all participants using an interview guide with open-ended questions. All interviews were held in Hebrew and took place through video-recording software (Zoom) due to the special circumstances of the COVID-19 pandemic. For each participant, we first collected demographic information that included age, marital status, number of children, and area of residence. We ceased recruitment when EB and LA agreed that thematic saturation had been achieved.

### Data analysis

A professional transcriber transcribed all the interviews verbatim. EB, LA, and AB read the interviews. At least two authors read each interview. We used thematic analysis to explore women’s attitudes and experiences with their PCPs regarding breastfeeding concerns. We followed the six-step framework for conducting thematic analysis suggested by Kiger and Varpio [7]. Each author read the transcribed interviews and familiarized himself or herself with the data. Next, each author independently marked statements regarding the aim of the study and assigned initial codes to each statement. Each author managed his or her codes in Excel. We than compared and grouped all codes into a single Excel sheet. We grouped all codes into 17 subthemes and later grouped these subthemes into four themes. We resolved disagreements through discussion. Next, we reviewed all themes and named them. After we agreed on the themes and subthemes, EB chose supporting quotes, and all the authors reviewed them together to determine whether they were suitable. The quotes were later translated into English by a professional translator.

## Results

Fifteen women in total expressed their interest in participating in this study. However, two were excluded following several failed attempts to reach them, so the final sample consisted of 13 women. All the interviews were conducted between December 2020 and May 2021. The interviews ranged from 23 to 61 min in duration, with an average of 41 min. All participants were interviewed in Hebrew.

Participants’ ages ranged from 24 to 37 years. All were married with a child aged two to six months. The children were the first, second, or third in the family. Women were all secular or religious, middle-class in terms of socioeconomic status, living in the southern region of Israel, and registered with MHS.

### Main themes

**Fig. 1:**
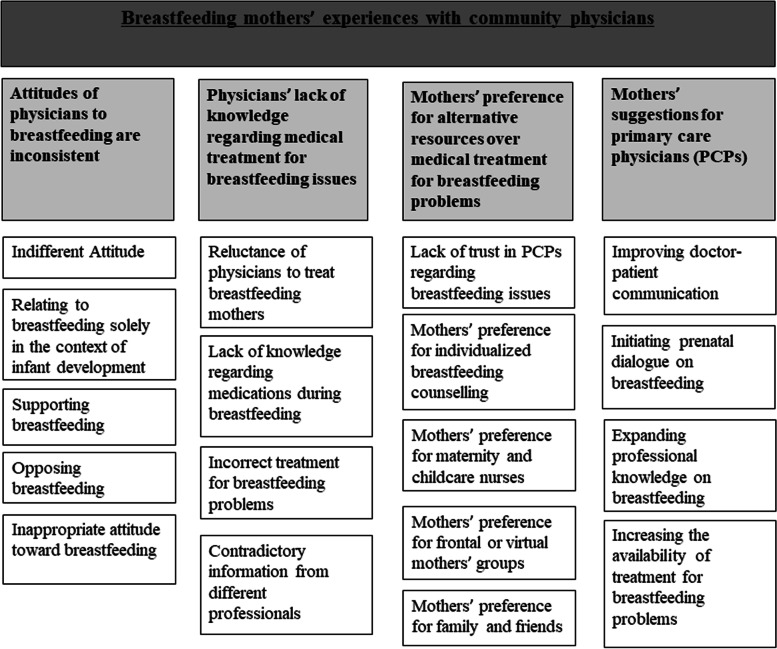
Themes and subthemes on the experiences of breastfeeding mothers with primary care physicians (PCPs) regarding breastfeeding

We identified four main themes: physicians’ inconsistent attitudes toward breastfeeding, physicians’ lack of knowledge regarding medical treatment for breastfeeding issues, mothers’ preference for alternative resources over medical treatment for breastfeeding problems, and mothers’ suggestions for PCPs. Each theme contains four to five subthemes (Fig. 1).

### Physicians’ inconsistent attitudes toward breastfeeding

For this theme, we identified five subthemes: indifferent attitude, relating to breastfeeding solely in the context of infant development, supporting breastfeeding, opposing breastfeeding, and inappropriate attitude toward breastfeeding.

#### Indifferent attitude

Women felt their physicians did not care whether they breastfed or not.

*‘[The doctor] just asked whether I was breastfeeding or not … She asked what the feeding method was, and as soon as I said exclusive breastfeeding, she never asked again.’ [id #6]*

*‘I think that they need to record the feeding method in the computer, so they always ask. So, every pediatrician always asks what the baby eats and how he eats… I never felt that it went beyond “what does the baby eat?’ [id #8]*

#### Relating to breastfeeding solely in the context of infant development

This subtheme was touched upon by almost all women. They felt physicians were interested in the way in which the child was fed only in the context of development and normal weight gain.

*‘I understood very quickly at the first meeting that he wasn’t necessarily a breastfeeding supporter… I think many doctors are like that. [The doctor] is in favor, very much, of child development, and it doesn’t matter what [the baby] eats, but what interests him is that the boy is developing properly. He said something like “even in pilot training they don’t check whether the child was nursed or not. As far as I’m concerned, baby formula is not a dirty word.”‘ [id #12]*

#### Supporting breastfeeding

Some women felt that some of their physicians, but not all of them, were supportive of breastfeeding.

*‘She said it was good and she encouraged it. In other words, she was very supportive. She said, “Great, good for you ... you’re breastfeeding … as long as it goes well, carry on, for sure.”‘ [id #7]*

#### Opposing breastfeeding

A minority of women felt that their physicians were opposed to breastfeeding. They pointed out that they were surprised to find that their physicians had such an attitude.

*‘When I got to the second doctor … right away his attitude was totally against breastfeeding … He said to me, “You know, you really don’t have to breastfeed. Even if they say that breastfeeding is better, it’s not necessarily proven.” He had an entire theory … [that] breast[milk] is not necessarily the preferred type of milk.’ [id #5]*

#### Inappropriate attitude toward breastfeeding

Two women revealed troubling stories during the interviews. Both felt that the physician’s behavior had been inappropriate. In the first case, the mother felt uncomfortable about the physician’s comments regarding her breasts.

*‘I once went, while nursing, for an annual breast check-up with a surgeon that I always go to. I sat across from him. I didn’t have a shirt on, and I said to him, “Oh, I’m nursing,” and he said, “Yes, I see”…What did he see there? God knows.’ [id #3]*

In the second case, the physician showed the woman how to express milk from her breasts. She felt comfortable with it, appreciated the help, and felt it was good advice for her. However, she sensed that this behavior may have been inappropriate for other women and unacceptable to them.

*‘He [a gynecologist] told me he had a few things to emphasize and … tell me so that the milk would be from the back and not the front. He … sat me down and showed me exactly what to do … how to massage the breast, really explained it all to me… he asked me if it was OK. Really, suddenly he showed me how the milk was coming out like, like a river...’ [id #11]*

### Physicians’ lack of knowledge regarding medical treatment for breastfeeding issues

We identified four subthemes of this theme: reluctance of physicians to treat breastfeeding mothers, lack of knowledge regarding medications during breastfeeding, incorrect treatment of breastfeeding problems, and contradictions among HCPs.

Women expressed their feelings that physicians provided insufficient medical treatment for breastfeeding issues. This situation was confusing and frustrating for women who were struggling with these problems and felt they could not receive proper treatment.

#### Reluctance of physicians to treat breastfeeding mothers

Several women were refused when attempting to make appointments with PCPs regarding their breastfeeding problems.

*‘I got blocked up right at the beginning and then it all cleared up and was fine. But after a few days, [my breast] really hurt. I was concerned that it was an infection or some kind of inflammation. My doctor was on maternity leave. I tried to contact another doctor… and [the clinic secretaries] told me that the [family] doctors couldn’t see me … because they didn’t have anything to examine or say.’ [id #10]*

#### Lack of knowledge regarding medications during breastfeeding

Many women felt that when they needed to receive medications while breastfeeding, they were unable to elicit suitable answers from their physicians regarding whether or not certain medications were contraindicated during breastfeeding and whether or not they could be harmful to their babies.

*‘I went to the family doctor, who … told me that there was nothing to be done about it, and that even in terms of painkillers there was not much that could be done because I was breastfeeding. Except for [paracetamol]. … [Etoricoxib] and stuff like that wasn’t relevant in my case. I felt like I wasn’t getting any … help.’ [id #4]*

Women often felt they had to search this information on their own (through the Teratology Telephone Center at the Ministry of Health).

*‘Also, when I checked which drugs could help, he said … maybe yes, maybe no… he said, “Maybe this, but you’re breastfeeding so I’m not sure it’s possible.” That was the dialogue. It’s like there’s some kind of vacuum. If I find myself searching on my own [for information on] whether I can take the drug or not, I think there’s a problem …’ [id #4]*

#### Incorrect treatment for breastfeeding problems

Another source of frustration for women was their sense that physicians were giving them incorrect treatment for their breastfeeding problems. A woman who came to request treatment for her baby’s oral thrush reported the following:

*‘I turned to one pediatrician, and he told me that all babies have yeast on their tongues, and it didn’t require treatment. The second one treated it but didn’t relate to me at all … she was very decisive and said, “this is definitely a yeast infection, let’s treat it,” but no one treated me.’ [id #3]*

When treating mastitis with antibiotics, physicians advised women to stop breastfeeding, as this excerpt shows:

*‘He told me that I had an infection and … needed an antibiotic. He said it automatically, even before he examined me. That I must take an antibiotic. And right away he said, “Because you need an antibiotic you can’t breastfeed him.” So, I asked him why I couldn’t breastfeed, and he said to me “because it can give the baby diarrhea if you have an infection.”‘ [id #10]*

In another example, a woman came to her physician and presented her plan to become pregnant while breastfeeding:

*‘He started to talk to me about birth control. I said, “No, we don’t need birth control because we want to become pregnant.” And then he said that it wasn’t good when you were breastfeeding. Both because you can’t become pregnant while breastfeeding, and also because it wasn’t healthy or something like that.’ [id #11]*

#### Contradictory information from different professionals

Women felt they had received conflicting information regarding correct breastfeeding behavior from various HCPs, especially physicians and nursing counsellors.

*‘I continued to breastfeed. But there were many conflicts. Basic things like nursing counsellors saying I should breastfeed on demand, when the baby wants it, at least … at the beginning. And [the physician] said to me, “That’s completely wrong, the baby needs to eat every three hours. If you feed him more often than every three hours, it causes gas.”‘ [id #12]*

### Mothers’ preference for alternative resources over medical treatment for breastfeeding problems

For this theme, we identified five subthemes: lack of trust in PCPs regarding breastfeeding issues, mothers’ preference for individualized breastfeeding counselling, mothers’ preference for maternity and childcare nurses, mothers’ preference for mothers’ groups (with virtual access or in-person meetings), and mothers’ preference for family and friends.

Most women reported that they preferred the above-mentioned alternatives to their physicians. It seems that lack of trust has led women to turn to breastfeeding counsellors, nurses, mothers’ groups, or family and friends rather than to their own physicians. Women reported that they trusted these people more than they did their physicians regarding breastfeeding advice and felt more comfortable with them.

#### Lack of trust in PCPs regarding breastfeeding issues

Most women in this study expressed their lack of trust in their physicians regarding breastfeeding issues. They felt that physicians lacked knowledge and tools with which to address these problems.

*‘It’s like grabbing someone in passing on the street, like involving the grocer in breastfeeding problems. It seems disconnected to me.’ (relating to the pediatrician) [id #11]*

*‘I really like my family doctor. I just didn’t feel that this was something that… I needed her for. I didn’t think it was relevant.’ [id #1]*

*‘I don’t really see a situation in which someone else can help me … not the family doctor, not the gynecologist.’ [id #4]*

#### Mothers’ preference for individualized breastfeeding counselling

Most women felt confident discussing their breastfeeding issues with individual breastfeeding counsellors. Most mentioned that they had met a counsellor immediately after delivery.

*‘If I was seeking advice, I might go to a breastfeeding counsellor, not necessarily to a doctor.’ [id #8]*

*‘The Maccabi breastfeeding counsellor, I invited her to my house, and she helped me. And in general, you can read a lot in Facebook groups on the internet about whatever is needed.’ [id #7]*

*‘So, straightaway I spoke to the breastfeeding counselor. That is, I also spoke to all the counsellors at the hospital, but a day later I already had a Maccabi breastfeeding counsellor.’ [id #6]*

#### Mothers’ preference for maternity and childcare nurses

Women in Israel are advised to see childcare nurses seven to ten days after delivery, and then again, every month or two. Most childcare nurses are qualified breastfeeding advisors or counsellors.

*‘They say that after three days you have to go to the maternity and childcare center, so I made an appointment. And she calmed me down. She sat me down physically … on the breastfeeding couch and showed me how to breastfeed with the silicon nipple.’ [id #11]*

*‘The Maccabi maternity and childcare centers are very, very encouraging. I didn’t feel tense at all. They encouraged me, gave me tools . . . more appropriate nursing schedules, and stuff like that.’ [id #1]*

#### Mothers’ preference for frontal or virtual mothers’ groups

Many women told us they attended mothers’ groups, such as La Leche League, to receive support and assistance with breastfeeding issues. There are also virtual groups on social media (especially Facebook) where mothers can share their difficulties and receive help from other mothers.

*‘When you give birth, there are all kinds of groups here. You’re already a mother for the third time, and you have friends, there are mothers from the nursery school. There are all kinds of groups whose advice at times helps more than medical opinion.’ [id #6]*

#### Mothers’ preference for family and friends

Other important sources of support and advice were women’s family and friends.

*‘I got a lot of support from the family. And … from friends who were also breastfeeding and said to each other “it’s OK, you’ll get through the first three weeks, you get past it. It’s hard at the beginning, it passes.” All kinds of things, you know, things that really helped me carry on … tips that I got from friends, and yes, in general, from other mothers.’ [id #12]*

### Mothers’ suggestions for primary care physicians

We identified four subthemes of this theme: improving doctor–patient communication, initiating prenatal dialogue on breastfeeding, expanding professional knowledge on breastfeeding, and increasing the availability of treatment for breastfeeding problems.

#### Improving doctor–patient communication

Women advised physicians to communicate more, be pleasant and polite, and take a genuine interest in breastfeeding issues. Physicians should emphasize the mother’s feelings (her mood). Another suggestion made by several women was not to pressure patients excessively about breastfeeding and to accept and support their decisions regarding whether or not to breastfeed.

*‘The doctor should be polite, nice. The communication should really be good; they should really take an interest and ask questions. It is really convenient for me if they are available over the Internet. That’s ideal ...’ [id #10]*

*‘It’s very important to show the whole picture. Every coin has two sides. [Doctors] do encourage [women] to breastfeed, and that’s good, that’s praiseworthy. But not every woman can breastfeed. And in the end, in my opinion, they do put a lot of emphasis on [breastfeeding], and some women can’t [breastfeed] and then, in the end, they feel bad about themselves that they can’t do it.’ [id #11]*

*‘A lot of attention [should be given] to how the mother feels, because I think that it has a great effect on breastfeeding. How she feels, how tired she is, what her mood is like, what the source of her support is. I think that these things have a great effect on breastfeeding.’ [id #9]*

#### Initiating prenatal dialogue on breastfeeding

Women suggested that physicians initiate dialogues on breastfeeding during the pregnancy to increase the likelihood of successful breastfeeding.

*‘If a woman comes prepared … the path to breastfeeding is much easier for her. If someone who is an authority, like her gynecologist, whom she trusts … throughout the pregnancy … [says something] like, “Sure, nurse the baby. Why not? That’s excellent,” he doesn’t have to say anything else. He doesn’t have to advise her on anything, just to support this thing.’ [id #3]*

#### Expanding professional knowledge on breastfeeding

Most women suggested that physicians expand their knowledge on breastfeeding so that they can provide good therapeutic options for women.

*‘Yes, I would be really happy to know whom I could turn to if my breasts were uncomfortable … if I had pain … or something like that. Because nobody actually is responsible for this, like medical breastfeeding. No one actually deals with it. Pediatricians only deal with this within the context of nutrition for babies.’ [id #6]*

*‘In a utopian world, doctors would be trained … even on the basic things that are common in the community. They don’t have to know the complex clinical things on breastfeeding. But at least the things that are seen in the community. Mastitis, and obstructed ducts, and yeast infections, and all these things … these are the difficulties … most women face.’ [id #3]*

*‘They should know the basic things, what to [prescribe]. Which antibiotic to give, which ointment … how to advise the woman… and, if necessary, they should refer [her] to a nursing counsellor … from the HMO, or to … professionals who know how to manage the things that they don’t know how to cope with.’* [id #3]

#### Increasing the availability of treatment for breastfeeding problems

Women suggested that physicians increase their appointment availability for the treatment of breastfeeding problems and lengthen their appointments.

*‘Pediatricians are OK, but you feel like they are always in a terrible rush … you feel as soon as you sit down that you have to get up.’ [id #6]*

## Discussion

This qualitative study explored the experiences and thoughts of mothers at appointments with PCPs that addressed breastfeeding issues. The themes that emerged concerned PCPs’ diverse attitudes towards breastfeeding, physicians’ lack of knowledge regarding medical treatment for breastfeeding issues, women’s lack of confidence in PCPs as capable of treating breastfeeding problems, preference for advice from other medical or social resources, and breastfeeding mothers’ suggestions for their physicians.

While many mothers experienced physicians as indifferent to breastfeeding or interested in it only in terms of the baby’s growth, some felt that physicians downplayed its importance. Physicians’ interest in breastfeeding was mainly described as formal and lacking depth. Cross-Barnet et al. revealed similar findings; approximately one-third of women in their study reported that pediatricians did not inquire about feeding at all. Another third reported that pediatricians did ask about feeding, but did not ask any further questions or provide additional information [8].

Most studies suggest that the majority of physicians support breastfeeding [9–11], and yet few PCPs think that their advice on breastfeeding is important [12]. Contrary to physicians’ feelings, many patients rated their doctors’ advice as very important [12].

We report two cases of mothers who felt PCPs’ treatment of their breastfeeding concerns was inappropriate. It is essential to treat mothers with respect for their bodies, as suggested in a study conducted by Blixt et al. [13].

The mothers in our study described experiences with physicians who refused to treat breastfeeding problems, recommended inappropriate medications, incorrectly advised mothers to stop breastfeeding because of the need to take antibiotics or other medications, lacked knowledge, provided inadequate or incorrect treatment of breastfeeding complications, and contradicted breastfeeding counselors’ advice. Dietrich Leurer and Misskey found that only 7% of mothers mentioned HCP as sources of helpful information on breastfeeding. They also revealed women’s experiences of receiving conflicting professional advice regarding breastfeeding management [14].

In line with our findings regarding physicians’ lack of skills and knowledge and conflicting advice, Holtzman et al. showed that PCPs had positive attitudes towards breastfeeding, but frequently lacked knowledge about it [10]. Almeida et al. found that HCPs perceived breastfeeding as an instinctive and natural biological act that did not require them to have any specific skills or sensitivity [15]. Dattilo et al. and Fox et al. found that many parents felt that the feeding advice of their HCP was often partial, inadequate, and inconsistent [4, 16]. Fox et al. also found that many women considered the advice they received from different HCPs about breastfeeding was often lacking and contradictory and undermined their confidence in their ability to breastfeed [16]. Moreover, a study conducted by Montalto et al. found that incorrect counseling by HCPs was the leading cause of breastfeeding cessation [17].

To overcome such contradictions between HCPs and improve PCPs’ knowledge and skills, communication and teamwork is crucial. Work in a multidisciplinary team is more effective than expert advice [18]. Most mothers prefer to turn to other sources, e.g., breastfeeding counsellors, mothers’ groups on social networks, close friends, and family. This theme is consistent with the results of the Israeli Ministry of Health’s infant nutrition survey, which found that less than half of mothers saw the doctor as a source of information about breastfeeding [5]. Similarly, Johnson et al. found that women expressed distrust of breastfeeding information received from HCPs and felt that they did not receive breastfeeding support when they needed it [19].

In our study, we found that women expected physicians to be able to discuss breastfeeding issues with them and treat problems appropriately. They wanted their physicians to be sensitive, patient-centered, and capable of tailoring their explanations to women’s needs. Some women thought it would have been helpful to receive information about breastfeeding during their routine pregnancy follow-up.

Previous studies on breastfeeding mothers’ experiences have yielded similar findings. Women advised HCPs to offer sensitive and individualized evidence-based breastfeeding support and medical care by creating a respectful dialogue [13]. Breastfeeding mothers valued HCPs who were supportive and non-judgmental and allowed them to make independent decisions [16].

The mothers’ suggestions are also supported by studies that have demonstrated the impact of physician’s support on breastfeeding initiation and duration. Mothers who went to PCPs who attended a breastfeeding education program were more likely to report exclusive breastfeeding at four weeks and longer breastfeeding duration and less likely to report any breastfeeding difficulties [20]. Several studies found that mothers were more likely to initiate and continue breastfeeding if their PCPs had encouraged them to breastfeed [21, 22].

The lack of support for mothers and treatment of breastfeeding problems by PCPs may be related to a gap in medical education and a failure to provide skills in the field of breastfeeding medicine. Gary et al. examined the curricula at US medical schools and found that breastfeeding medicine education is lacking and that students are uncomfortable with guidelines for breastfeeding support and basic treatment [23]. A study by Meek et al. revealed gaps in physicians’ basic knowledge and clinical skills in supporting breastfeeding. Moreover, physicians would like to receive additional breastfeeding education as part of their medical training [24]. Holms et al. showed that physicians who participated in a breastfeeding education program increased their professional knowledge and their patients’ breastfeeding rates rose [25]. We therefore believe that in order to practically apply the results of the study and provide appropriate medical care to patients who are breastfeeding mothers, breastfeeding education programs should be integrated into the curricula of medical schools; internship programs in gynecology, pediatrics, and family medicine; and professional training for physicians. Such training programs for students have demonstrated improved knowledge and attitudes, better breastfeeding support skills, and improved self-confidence [26].

### Strengths and limitations of this study

The strengths of this study are that it provides an in-depth qualitative examination of women’s perceptions of their encounters with their PCPs related to breastfeeding. The limitation of the study is its relatively small sample size. However, because we reached data saturation, the sample is considered sufficient. In addition, the sample may be prone to selection bias; the women participants were all Jewish, secular, educated, and middle-class. Women from these groups may be more likely to initiate breastfeeding and more willing to participate in study interviews. They may also be more able to make judgments regarding their own health needs and may have the ability to seek out support in the community as well as through the internet. Another possibility is that these women may be more assertive with their PCPs in requesting what they need, more capable of seeking out community support, and, as a result, also more likely to identify contradictions and lack of knowledge in PCPs.

## Conclusions

In this study, we focused specifically on PCPs – community physicians, family physicians, pediatricians, and gynecologists—who serve as breastfeeding women’s primary physicians. As such, they should be able to provide professional and tailored medical care to address concerns regarding breastfeeding management and infant weight gain, prescribe appropriate medications to breastfeeding patients, and manage common breastfeeding problems. The lack of trust the participants in this study expressed towards PCPs regarding breastfeeding problems was profound; they reported insufficient breastfeeding support by PCPs and incorrect or inadequate treatment of medical problems they experienced while breastfeeding. They also reported feeling they had no medical expert to approach with breastfeeding-related problems. We believe that physicians should expand their knowledge on breastfeeding medicine so that they can provide comprehensive patient-centered medical treatment to both mothers and children. Education programs for improving knowledge and skills in breastfeeding issues should be implemented throughout the medical training; starting from medical students, through syllabus of medical residents and scheduled workshops for specialists in different fields.

## Data Availability

The transcribed interviews analyzed during the current study are not publicly available for reasons concerning the interviewees’ privacy but are available from the corresponding author on reasonable request.

## Abbreviations

HCPs: Health care providers
HMOs: Healthcare maintenance organizations
MHS: Maccabi Healthcare Services
PCPs: Primary care physician
